# Shared and Supported Decision Making in Medication in a Mental Health Setting: How Far Have We Come?

**DOI:** 10.1007/s10597-021-00780-2

**Published:** 2021-02-05

**Authors:** Sofia Pappa, Joshua Barnett, Sally Gomme, Anthi Iliopoulou, Ivan Moore, Michael Whitaker, Jane McGrath, Michele Sie

**Affiliations:** 1 West London NHS Trust, London, UK; 2 Dept of Psychiatry, Imperial College London, London, UK; 3 We Coproduce CIC, London, UK

**Keywords:** Shared decision-making, Supported decision-making, Medication choice, Mental health, Co-production

## Abstract

Personalised care involves shared decision making (SDM) across all levels including choice in medication. However, there are a number of barriers which prevent its effective implementation in routine mental health settings. Therefore, we undertook a study to benchmark current practice across clinical services of a large urban mental health provider. The study formed part of the trust-wide ‘Supported Decision Making in Medication’ Co-Production Project and aims to inform future recommendations in delivering against contemporary best practice, guidance and policy. A survey exploring the views and experiences of service users and prescribers on shared and supported decision-making in medication was carried out in West London NHS Trust. Questionnaires were fully co-designed and co-delivered by a group of health professionals and individuals with lived experience. There were 100 responses from service users and 35 from prescribers. There was some good practice where both parties reported good quality conversations concerning dialogic styles, collaborative process, information provided and range of choice offered. However, prescriber’s perception of their practice was not always mirrored by service user feedback whose experiences often depended upon the prescriber, the time available or the part of the service. Generally, service user experience fell short of the good practice cited by clinicians though there was noticeable variability. Commitment from organizations and increasing understanding from practitioners are vital in transforming SDM from rhetoric into reality. From our findings a further challenge is to ensure that prescribers and service users have the time, information and tools to implement it consistently.

## Introduction

Clinical decision-making is a complex process that sits at the heart of mental health service delivery. Three levels of patient involvement in decision-making have been described: shared, supported and passive. Shared decision-making (SDM) is collaborative decision-making involving the sharing of information and expertise valued by both participants (Slade 2017). It is a process in which individuals are supported by clinicians to understand and jointly decide what support and/or treatment is the most suitable bearing in mind the person’s own preferences, culture and circumstances. Passive or clinician-led decision-making occurs when the clinician makes the decision for the patient while in supported or patient-led decision-making the individual makes a choice after receiving advice and information from a professional and other reliable sources (Bach 2006; Stavert 2016).

SDM is considered the golden standard of modern healthcare provision (Tse 2017). The NHS Five Year Forward View set out a vision of a new relationship between people and professionals, and between the care system and communities (NHS 2016). The new relationship should be based on partnership, and an understanding that expertise and experience do not rest only with professionals or organizations but also with individual patients. This was further reinforced by the NHS Long Term Plan: ‘Since individuals’ values and preferences differ, ensuring choice and sharing control can meaningfully improve care outcomes. Creating genuine partnerships requires professionals to work differently, as well as a systematic approach to engaging patients in decisions about their health and wellbeing.’ (NHS 2019).

Although, evidence thus far appears inconclusive on whether or not effective use of SDM translates to better clinical outcomes, it clearly increases patient satisfaction (Silva 2012). The CEDAR study (Puscher et al. 2010) showed that both patients and clinicians had a preference for SDM and that patients felt “disempowered” whenever they perceived that they had less involvement in a decision than they would have liked. Similarly, recent studies and systematic reviews confirm that patients who actively participate in shared decision making experience higher satisfaction with their care (Shay and Lafata 2015; Einterz et al. 2014) and less decisional conflict or challenge to personal values (LeBlanc et al. 2009).

Despite this, it seems as though effective implementation of SDM by mental health services in the UK has been inconsistent, particularly with regards to decision making about medication. In the CQC NHS Community Mental Health Survey in 2019 (Quality and Commission 2019) respondents were asked if they were involved as much as they wanted to be in decisions about their medications. 51% of people responded ‘yes, definitely’, 38% responded ‘yes, to some extent’ and 12% responded ‘no’. Likewise, a report by the charity Rethink (2006) showed that many patients reported having limited information and choice when a prescribing decision was made. Furthermore, the MAGIC program (2017) found that clinicians were often of the opinion that they already involved patients in decisions about their treatment and therefore not able to see the difference between their practice and SDM or were of the opinion that patients often did not want SDM; they also reported having too many other demands on their time to contend with (Joseph-Williams 2017).

The aim of this cross-sectional study was to evaluate the types and level of collaborative decision making in medication taking place across clinical services in West London NHS Trust (WLHT, a large urban mental health care provider) and compare patient experiences and prescribers’ self-reported practice against best practice. Findings would further allow to identify potential barriers and inform future recommendations to encourage collaborative decision making in clinical encounters involving a change in medication.

## Methods

### Prescriber and Service User Survey

The questionnaires for both cohorts (prescriber/service user) were collaboratively constructed by the ‘Supported Decision Making in Medication Project’ co-production group, a partnership between West London NHS Trust and We Coproduce, a West London Collaborative. Co-production in healthcare means that patients contribute to a project such as service development or research as equal partners of professional providers. As such, the core group consisted of both service users with lived experience and professionals including a chief pharmacist, a consultant psychiatrist, the WeCoproduce CEO and a wellbeing network team leader. Ethical approval for this study was granted by West London NHS Trust.

Survey questions were produced by the group, with much debate around language, tone and assumptions. When differences of opinion occurred, the opposing views would be discussed until a consensus was reached; where this was not possible the majority view was adopted. The questions for both surveys were designed to inform upon following topics: preferred decision making style, choice given to patients in regards to their own treatment, information provided regarding medication including side effects, the role of potential time pressures, exit strategy and alternative treatment options offered. Although there is significant overlap between the two surveys, questions and answers are not identical and differences aim to capture characteristics and experiences specific to each group (Appendix). The majority of questions in the prescribers’ survey used a five-point scale to record responses, whereas similar questions in the service user survey did not always use identical responses. Despite these differences, the surveys allowed for the topic concepts to suitably align. The survey for prescribers contained 21 questions and for service users 34 questions. Both surveys included a comments section to allow users to explain their rationale or add further detail to their answers if needed.

### Participants and Setting

The online survey was sent via email to all prescribers within the Trust and was open to clinicians working both in inpatient and community services. The service user survey took place during the same period of time and was mainly conducted in person in community mental health teams by peer members of We Coproduce who approached patients attending for outpatient appointments. In addition, the survey was available online through the Trust website and was also disseminated to existing service user groups and forums within the Trust. Participants for both surveys were self-selected and their responses remained anonymous; there was no requirement for a therapeutic connection between the prescribers and the service users who took part in the respective surveys.

### Data Analysis

Simple descriptive and comparative statistical tests were performed on the aggregated anonymised data by members of the study group.

## Results

**Fig. 1:**
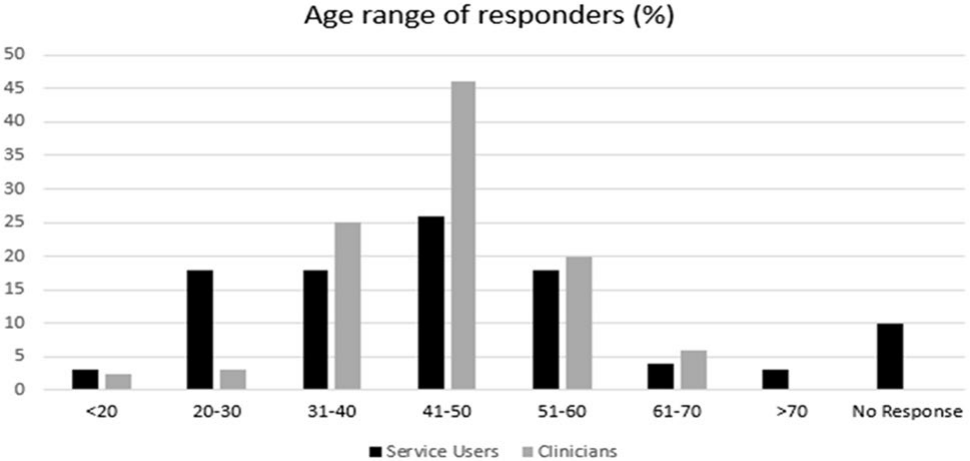
Age range of service users and prescribers

There were 35 responses to the prescriber questionnaire with a response rate of 40% (35/87). 49% of clinicians who provided answers worked in an inpatient setting and the remaining 51% worked in outpatients. The majority of clinicians (64%), were consultant grade, 12% were higher trainees and the remainder (24%) were junior trainees. There were 100 responses to the service user questionnaire. The ethnicity of the service users was as follows: White 50%, Black 10%, Asian 16%, Mixed Race 11%, Arabic 2% and 11% did not wish to provide this information. The age range among amongst responders can be seen in Fig. 1; the majority of responders fell within the same age range 41–50 in both groups.

As the focus of the survey was to compare service users and prescriber’s views and experiences in relation to medication choice, relevant results from both surveys are presented below grouped together or next to each other for ease of reading and where useful. However, the two groups are not always asked the same question and in some cases direct comparisons are not possible across the two cohorts.

### Decision Making Style Preferences and the Collaborative Process

**Fig. 2:**
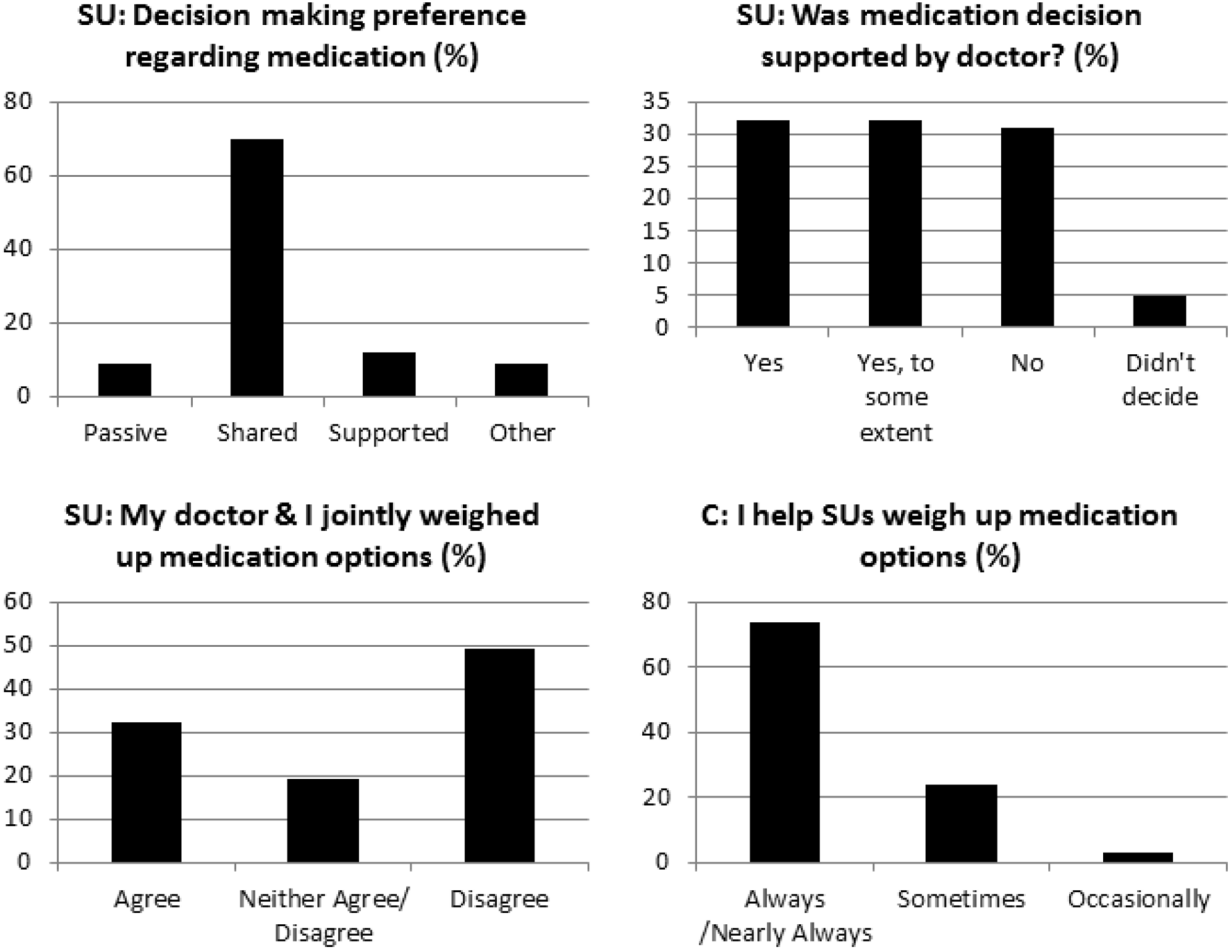
Desired and actual decision making style and how clinicians perceived their own practice vs. how it was experienced by SUs

As shown in Fig. 2, the majority of service users (70%) stated that they preferred shared decision-making regarding their medication (45% with their doctor only, 25% with their doctor and family) while 12% wanted to make the decision themselves but with their doctor’s support (supported or patient-led decision making) and only 9% wanted their doctor to make the decision for them (passive or clinician-led decision-making). In practice, 32% said their decision was supported by their doctor and another 32% said their decision was supported to some degree while 31% said they did not make the decision while 5% didn’t want to decide (Fig. 2).

There was a clear difference between how clinicians and service users perceived the decision making process during a consultation: 71% of clinicians reported that they always or nearly always help service users to weight up different treatment options, 26% sometimes and only 3% occasionally (Fig. 2). However, nearly half (49%) of the service users disagree or strongly disagree with the statement “My doctor and I jointly weighed up the different medication options” and only 32% agreed/strongly agreed with this statement; the remaining 19% neither agreed nor disagreed (Fig. 2).

**Fig. 3:**
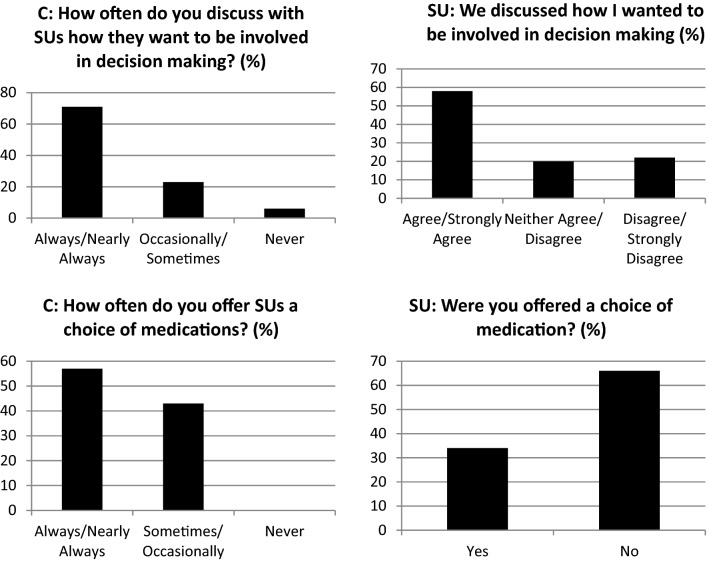
The incongruence between clinicians and service users with respect to the collaborative process

Furthermore, as indicated in Fig. 3 the majority of clinicians (71%) stated that they always or nearly always discuss with patients how they would like to be involved in decision making about medication with 58% of service users agreeing with this. The remaining 29% of clinicians reported that they sometimes, occasionally or have never had this discussion with patients. In addition, 57% of clinicians stated they always or nearly always offer a choice to their patients and 43% saying this happens only sometimes or occasionally. However, only 34% of service users stated that they were offered a choice of medications, with the remaining 66% responding that no choice was offered (Fig. 3). Clinicians indicated that they may deny or limit choice due to their perception of a patient’s risk; lack of capacity; the patient being in a forensic setting and alternative options being clinically inappropriate.

### Drug Information Provision

**Fig. 4:**
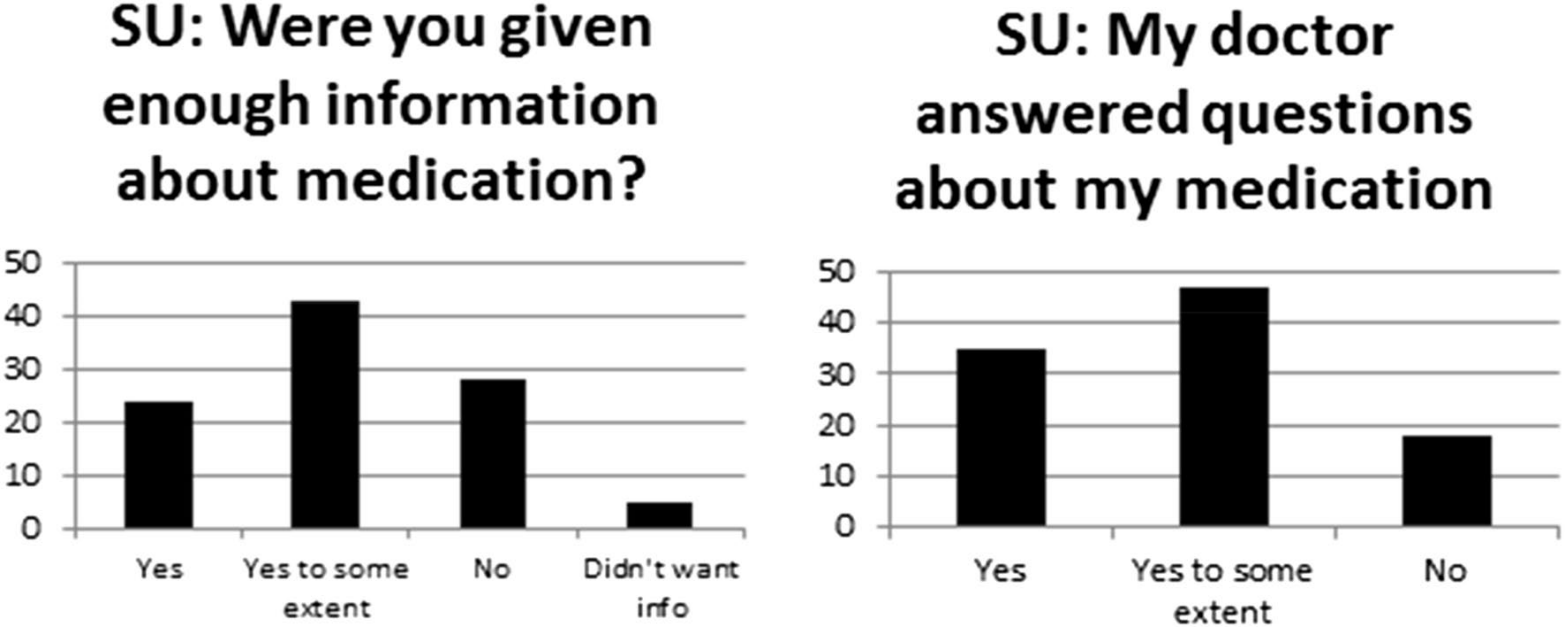
Information about medication provided by prescribers

Service users appear 'under-informed’ with regards to their medication. Only 24% of service users stated they received enough information about their medication (Fig. 4). The majority (59%) stated they did not receive enough information or received information to some extent on this matter.

When giving service users information about medications, a third of clinicians indicated that they relay this information verbally, 30% used the ‘Choice and Medication’ Leaflet, 24% used other leaflets and 13% used other internet resources. It is noteworthy that only 22% of service users stated that the written information they were provided was easy to understand.

Most clinicians reported explaining the purpose (94%) and therapeutic effects of medication (92%) while 42% of service users agreed their clinician told them what they thought the medication would help them with and a further 42% responding this only happened to some extent. Most clinicians reported explaining the benefits and risks of taking medications (89%), while 28% of service users said no explanation was given to them at all, 39% of service users said this was explained to some extent but only 33% of service users agreed that the benefits and risks of taking medication were definitely discussed (data not shown in graph).

**Fig. 5:**
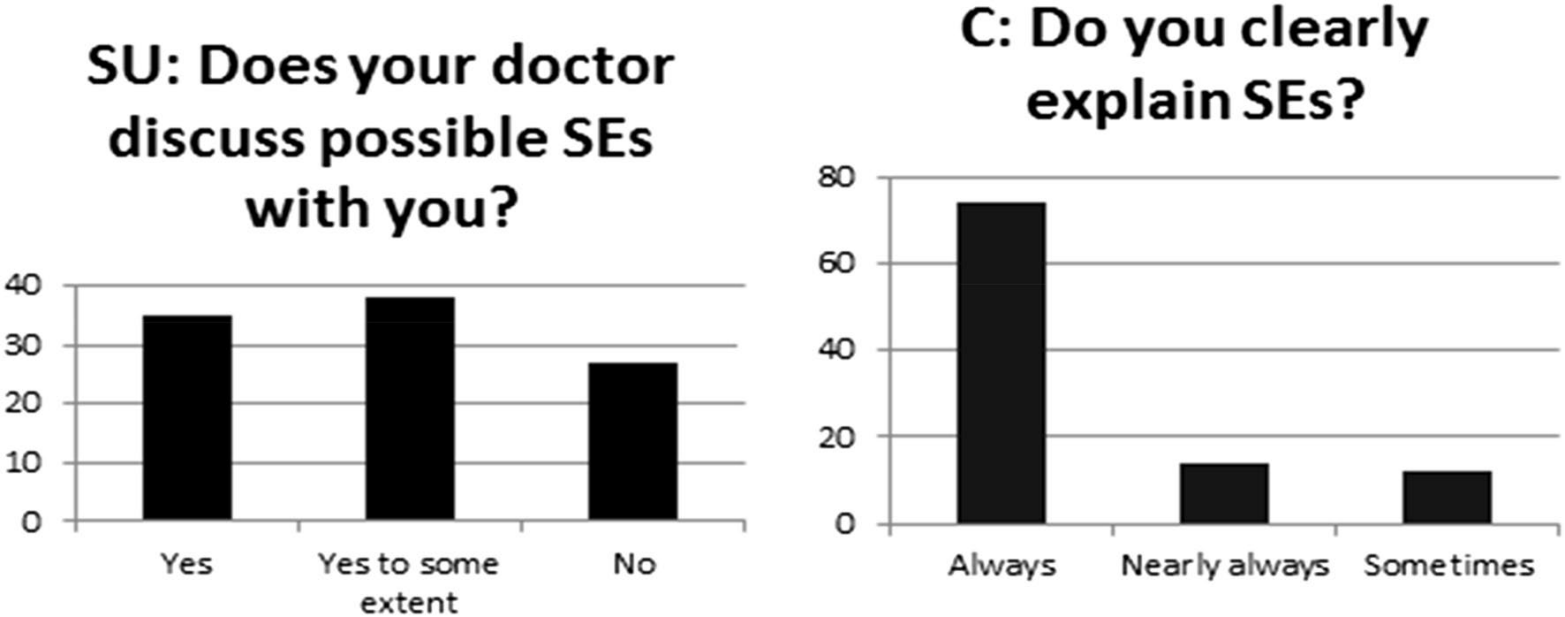
Information regarding potential side effects

On the topic of side effects (SEs) being explained to service users, 35% agreed that their doctor fully discussed this, 38% said this was discussed to some extent and 27% said this was not discussed at all as shown in Fig. 5. Contrarily, 88% of clinicians stated that they always (74%), or nearly always (14%), clearly explain potential adverse effects to their patients and only 12% reported that they only did so sometimes (Fig. 5).

### Time Pressures, Exit Strategy and Broader Treatment Options

**Fig. 6:**
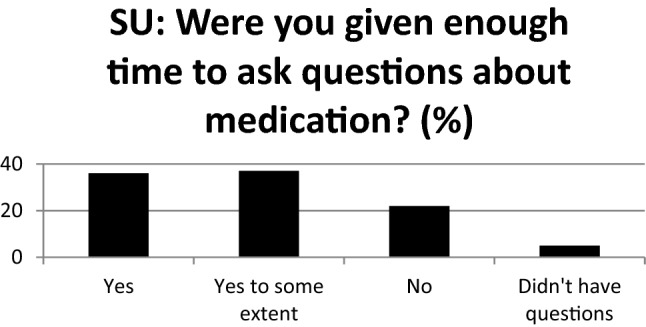
Time available to fully discuss medication

The majority of service users (59%) said they weren’t given enough time to fully ask questions about medication, with only 36% agreeing that they had (Fig. 6). Likewise, service users responded that the time pressure on clinicians prevented them from discussing reducing or stopping medication with only 21% having had conversations about coming off medication. Clinicians also reported that time constraints limited their ability to discuss duration of treatment and reducing or stopping medication.

**Fig. 7:**
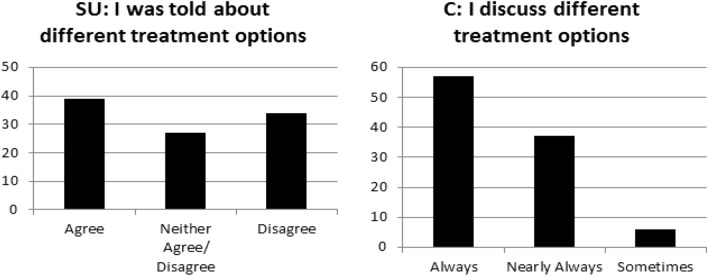
SUs and Clinicians’ responses regarding different treatment options offered

Most clinicians (94%) responded that they were discussing different treatment options all of the time or nearly all of the time but this was not the experience of the service users with over half (56%) stating that medication was the only option offered (Fig. 7). Most clinicians (60%) wanted to be able to offer broader, non-pharmacological treatment options e.g. social prescribing; social and vocational support; work-based daily therapy and therapeutic communities, family interventions and more rapid access to talking therapies.

## Discussion

Overall, a substantial proportion of prescribers at WLHT reported supporting best practice required around collaborative decision making in medication, information provided and range of choice offered. However, there is a lack of consistency in current practice, with patients reporting variable experiences around how decisions are made about their medication. Furthermore, there is significant incongruence around the quality of the reported conversations with prescribers assuming that more is being delivered/understood than service users indicated in their responses. These findings are similar to previous reports showing that, despite shared decision making being a widely accepted standard of patient-centered care (Morant et al. 2016), it is commonly underutilized in the field of mental health (Stead et al. 2017).

### Collaborative Process and Decision-Making Style

There are significant discrepancies between reported service user experience and the perception of clinical practice by the doctors in terms of preferred and actual decision making style. For example, the majority of patients responded that they would like to make decisions jointly with or supported by their doctor but only just over half of them reported that they were asked how they wanted to collaborate in the process.

The reasons for this disparity is likely multifactorial. Clinicians may be lacking time, information and decision making tools. It could be also that they are not fully aware of how SMD should be implemented or may think that patients don’t want it (Joseph-Williams 2017). However, this is not consistent with existing evidence showing that authentic experiences of patient-centred care and shared decision making were associated with positive experiences of care within mental health hospital settings (Borge and Hummelvoll 2008; Cleary et al. 2003, Staniszewska et al. 2019). In addition, the implementation of a shared decision making training program in a community setting was able to significantly change patients’ decisional conflict (Ramon et al. 2017).

### The Under-Informed Patient

The majority of patients responded that they didn’t receive adequate information regarding medication and potential side effects with a combined 65% answering that they were “not at all informed—to some extent informed”. The clinician’s role in sharing information is essential to enabling conversations with patients that make it possible for them to weigh up their options, so that shared and supported decision making is actually attainable. What is more, only 22% of service users stated that the written information they were provided was easy to understand. This is despite the Trust’s subscription to the ‘Choice and Medication’ website that provides easy to read leaflets on numerous medications and mental health conditions.

### Time Pressures, De-prescribing and Broader Treatment Options

There is a further concern around how much therapeutic time is available to have conversations in which questions can be asked and responses explored. Both patients and clinicians felt they lacked enough time in consultations to either ask or answer questions about medication including side effects and duration of treatment. In an effort to address time constraints, a previous study reported successfully trialing pee- led clinics in waiting rooms that offered opportunities for service users to explore with specialist peer workers the conversations that they want to have/ have just had with their doctors (Deegan 2010).

Involving patients more closely in the decisions about psychiatric medication has been also suggested as a means of reducing excessive prescribing (Brown and Bussell 2011). However, only a fifth of service users reported having a conversation about stopping medication. This together with a lack of discussion about alternative treatment options may lead clinical services to an over-reliance on long-term pharmacological approaches and with too little regard for de-prescribing (Morrison et al. 2012).

### Co-production Process

A major strength of the study is the fact that it has been fully co-produced by professionals and service users throughout its different stages from inception to completion. Members of this group were equal partners throughout the whole process: ideas generation, design, management, data collection, analysis, evaluation and dissemination. Patient and Public Involvement (PPI) is defined as service planning and delivery as well as research and quality improvement with and by patients, rather than to, for or about them (Staley 2009; The BMJ). In recent years, there has been a significant shift in PPI in all areas, moving away from tokenism, towards more meaningful involvement and co-production (Ocloo and Matthews 2016). Whilst some studies have reported on co-produced aspects of larger projects (Gillard et al. 2012), and have found positive impact on findings, there is generally limited evidence of genuine equal partnership where power is shared throughout project stages, particularly in data collection and analysis (Green et al. 2020; Dewa et al. 2019,2020). NIHR’s INVOLVE guidance on co-producing a research project focuses on achieving five main principles including sharing power, including all perspectives and skills, respecting and valuing the knowledge of all those working together, reciprocity and building and maintaining relationships (Hickey et al. 2018; Hickey 2018).

### Limitations

There are some limitations to the study which should be mentioned. The prescriber participants are drawn from different areas across WLHT, which may influence their practice. The survey was open to prescribers working in forensic and non-forensic inpatient and community services whereas the majority of patients surveyed came from a community setting although patients may have moved across services. Furthermore, patients experiencing mental health difficulties but looked after in primary care were not included which may have been particularly interesting with regards to de-prescribing experiences. Participating prescribers and patients were not necessarily therapeutically connected and respective questionnaires, albeit similar, were not identical. This makes it difficult at times to accurately compare the two surveys. Self-selection may have led those with an interest to take part. The size of the partaking cohorts means this is a snapshot of experience at a particular time. In addition, the lack of understanding of how representational the sample is (in the absence of a service user survey response rate) may limit the generalizability and accuracy of some of the findings.

### Future Steps

Whilst this study provides a small insight at a particular moment in time, it offers clinical services the opportunity to reflect where the decision-making power around medication really lies and how compliant they are in delivering against contemporary best practice, guidance and policy. Given the emphasis put on patient centred care, a key question for mental health care providers to reflect on is as to whether service users would state their preferences more clearly, if they were asked how they would like to be involved, given information they understood, had longer discussions and had peers or advocates to better support them. The above observed disparity may lead to explaining power relationships, dissatisfaction and most of all a resistance to culture change and organisations should not just see this as a problem but a potential open door for problem solving. Indeed, commitment from organizations and practitioners is key in transforming SDM from rhetoric into reality (Tse 2017) and increasing understanding of shared and supported decision making is an essential step in effective implementation (Lloyd et al. 2013; May and Finch 2009). Moving forward, organisations are therefore required to think more carefully about how they communicate important information regarding the right of the individual to be part of the decision making process and the choices available to them as one size will not fit all.

### Peer Training

Peer training at different levels and settings can be helpful. It was noticed, for example, that interactive SDM training workshops and role-play based training helped build an understanding of what SDM is, improved SDM skills (Burton et al. 2010) and also promoted positive attitudes towards the process in the MAGIC project (Hickey 2018). A key finding from the study was that whilst Decision Support Tools are useful “skills trump tools, but attitudes trump skills”. Embedding training on SDM in the under- and post-graduate training for clinicians is another obvious area for improvement.

### Follow-up Survey

A follow-up survey seeking the opinions and experiences of clinicians and patients in a variety of settings could be useful in discovering barriers that may be specific to different services. Focused work with clinicians and patients who are therapeutically connected may also help understand potential incongruences; targeted interventions could be put into place to address these discordances.

### Decision Support Tools

Decision support tools (DSTs) are innovative technologies that are used to help patients choose different treatment options (Henshall 2017) and can help to promote SDM by providing continuously updated information about medications. The use of DSTs has shown potential in terms of patient empowerment and increased awareness of possible side effects (Silva 2012; Leucht 2006; Kaar et al. 2019). The DST developed by Henshall et al. in 2019 (Henshall et al. 2019) allowed for patients to avoid medications which were more likely to cause the one side effect they most wanted to avoid (May and Finch 2009; Joosten et al. 2009; Novick et al. 2010; Morken et al. 2008).

## Conclusions

Personalized care involves collaborative decision making across all levels including choice in medication which requires a shared understanding of the ways of working and available options explained. A good outcome will come from a relationship where both parties are willing to share information and accept shared responsibility for joint decision-making; this may only be a subtle change of practice for some, but it could feel like a dramatic one for others. Our findings support the need to ensure that prescribers and service users have the time, information and tools to implement this change consistently.

## Appendix

### Prescriber Survey

All questions allowed for additional comments to be made as part of the answer.

1. I discuss with service users whether or not a decision needs to be made about medication at this time.

Always.

Nearly always.

Sometimes.

Occasionally.

Never.

2. I discuss with service users how they want to be involved in making decisions about treatment.

Always.

Nearly always.

Sometimes.

Occasionally.

Never.

3. I discuss with service users about the different treatment options available

Always.

Nearly always.

Sometimes.

Occasionally.

Never.

4. I explain the advantages and disadvantages of the different treatment options available

Always.

Nearly always.

Sometimes.

Occasionally.

Never.

5. I discuss with services users my opinion of the benefits and risks of taking medication and not taking medication (two part question, table available to answer each part separately)

Always.

Nearly always.

Sometimes.

Occasionally.

Never.

6. I offer service users a choice of medicines

Always.

Nearly always.

Sometimes.

Occasionally.

Never.

7. When discussing medicine I clearly explain the following: Purpose, Therapeutic Effects, Adverse Effects, Recommended Duration of Treatment (four part question, table available to answer each part separately)

Always.

Nearly always.

Sometimes.

Occasionally.

Never.

8. I help the service user to thoroughly weigh the different treatment options

Always.

Nearly always.

Sometimes.

Occasionally.

Never.

9. I ask service users which treatment option they would prefer

Always.

Nearly always.

Sometimes.

Occasionally.

Never.

10. I support the service user to choose their preferred treatment option

Always.

Nearly always.

Sometimes.

Occasionally.

Never.

11. Do you feel your patients are given enough involvement in decisions about which medications you prescribe?

Always.

Nearly always.

Sometimes.

Occasionally.

Never.

12. When a person on depot/long acting injection wishes to change to oral medication do you prescribe in accordance with their wishes?

Always.

Nearly always.

Sometimes.

Occasionally.

Never.

13. How do you support patients who wish to reduce or come off medication?

Please comment.

14. What information about the medicines do you provide to service users?

Verbal.

Leaflet—Choice and Medication.

Leaflet—Trust produced.

Leaflet—Other.

Internet resources.

15. Would you like broader treatment options to be available for you to offer?

Yes- if so, what? Add comments.

No.

16. Do you feel your choices for prescribing medicines are restricted?

Yes- if so, for what reasons? Add comments.

No.

17. How do you keep informed of the latest treatment options beyond medicines?

Please comment.

18. What non-pharmacological treatments are you aware of?

Please comment.

19. Which type of team do you mainly work in?

Inpatient.

Recovery.

## EIS

Other (please specify).

20. What grade are you?

Please comment.

TextBreak="Yes"21. What is your age?

 < 20.

20–30

31–40

41–50

51–60

61–70

 > 70.

### Service User Survey

1. Have you ever discussed medication with a West London Mental Health Trust doctor?

Yes.

No.

2. Are you currently taking medication to manage a mental health condition?

Yes.

No.

3. When did you last have a consultation where medication was discussed at West London Mental Health Trust?

In the last month.

2—6 months ago.

More than 6 months ago (if so please tell us how long ago in the comments box).

I have not had a discussion about medication.

4. In my discussion with my doctor we discussed exactly how I want to be involved in making decisions about medication

Completely Disagree.

Disagree.

Neither Agree or Disagree.

Agree Completely Agree.

5. How much involvement did you want to have in decisions around your medicines?

No Involvement (doctor makes the decision for me).

Shared Decision (joint decision with me and my doctor).

Shared Decision (joint decision with me, my family and my doctor).

Supported Decision (I wanted to make the decision myself, supported by the doctor).

Other (please specify).

6. My doctor told me that there are a range of different treatment options for my condition

Completely Disagree.

Disagree.

Neither Agree or Disagree.

Agree Completely Agree.

7. Was medication the only treatment option offered?

Yes.

No.

If no, what other options were offered? (Leave Comment).

8. My doctor gave his professional opinion of the advantages and disadvantages of medication as a treatment option

Completely Disagree.

Disagree.

Neither Agree or Disagree.

Agree Completely Agree.

9. My doctor clearly explained about the possible benefits and risks of not taking medication

Yes, definitely.

Yes, to some extent.

No.

10. How do you take your current medication?

Orally.

By injection.

11. Were you given a choice on using oral or injectable medication?

Yes.

No.

12. Were the advantages and disadvantages of both discussed?

Yes.

No.

13. My doctor supported me to understand all the information

Completely Disagree.

Disagree.

Neither Agree or Disagree.

Agree Completely Agree.

14. I was given enough time to ask questions about medication

Yes, definitely.

Yes, to some extent.

No.

I did not have any questions about medicines.

15. My doctor answered questions I had about medication

Yes, definitely.

Yes, to some extent.

No.

16. My doctor discussed what they felt the medication would help with.

Yes, definitely.

Yes, to some extent.

No.

17. My doctor discussed the possible side-effects of my medicines

Yes, definitely.

Yes, to some extent.

No.

18. My doctor discussed with me how long they felt I might need to take medication for

Yes, definitely.

Yes, to some extent.

No.

19. Did you and your doctor discuss future possibilities in terms of medication? Particularly:

Reducing medication.

Coming off medication.

Continuing to take medication.

20. Were you offered a choice of medicines?

Yes.

No.

If yes what options were offered?

21. My doctor and I jointly weighed up the different medication options

Completely Disagree.

Disagree.

Neither Agree or Disagree.

Agree Completely Agree.

22. Do you feel you made the decision about medicines supported by your doctor?

Yes, definitely.

Yes, to some extent.

No.

I did not want to make the decision. Can you tell us why? (Leave Comment).

23. Do you feel you got the treatment option that you wanted?

Yes, definitely.

Yes, to some extent.

No- if so, what would you have preferred? (Leave Comment).

24. If you decided not to take medication was your choice respected?

Yes, definitely.

Yes, to some extent.

No.

25. Are you prescribed medicines that you don't take?

Yes.

No.

26. Do you feel you were given enough information about the medicines you received?

Yes, definitely.

Yes, to some extent.

No.

I did not want any information about the medicines.

If you were given information please can you tell us what you received? (Leave Comment).

27. Were you given written information (e.g. leaflets) about the medicines?

Yes.

No.

28. Did you find the information easy to understand?

Yes, definitely.

Yes, to some extent.

No.

I was not given any information.

29. Where do you get information from to make decision about medicines?

Doctor.

Hospital Pharmacist.

Nurse.

Social Worker.

Community Pharmacist.

Occupational Therapist.

Care co-ordinator.

Charity.

Family.

Carer.

Other service user.

Internet.

Books.

Other (please specify).
